# Supplementary material for: P-TRAP: a Panicle Trait Phenotyping tool
Source: BMC Plant Biol. 2013 Aug 29;13:122. doi: 10.1186/1471-2229-13-122 (PMC3848748; doi:10.1186/1471-2229-13-122)
Supplement: Additional file 7 — P-TRAP source code. The Netbeans project that contains the source code of the application. [file 1471-2229-13-122-S7.zip › javadoc.zip!/javadoc/org-biolag-particleprocessor/org/biolag/particleprocessor/package-summary.html]

org.biolag.particleprocessor (ParticleProcessor)


JavaScript is disabled on your browser.


- Package
- Class
- Tree
- Deprecated
- Index
- Help

*org.biolag.particleprocessor 1.0*

- Prev Package
- Next Package

- Frames
- No Frames

- All Classes

# Package org.biolag.particleprocessor

- Class Summary

  | Class | Description |
  | --- | --- |
  |  |  |
  | --- | --- |
  | ConcavePoint |  |
  | Moments | The moments manipulator class. |
  | Particle | A general class for managing particles. |
  | ParticleFinder | The detector of the particles in binary images. |
  | ParticleProcessor | Is a set of algorithm for detecting particles on grayscale and colored images. |
  | ParticlesAnalyizer | Given a set of particles detected on a binary image, this class is responsible for analyzing them. |
  | ParticlesModel | A model class for I/O manipulating of the particles. |
- Enum Summary

  | Enum | Description |
  | --- | --- |
  |  |  |
  | --- | --- |
  | Particle.CONTOUR\_TYPE |  |
  | ParticlesAnalyizer.CALC\_METHOD |  |

- Package
- Class
- Tree
- Deprecated
- Index
- Help

*org.biolag.particleprocessor 1.0*

- Prev Package
- Next Package

- Frames
- No Frames

- All Classes
